# Novel Conductive and Redox-Active Molecularly Imprinted Polymer for Direct Quantification of Perfluorooctanoic Acid

**DOI:** 10.1021/acs.estlett.4c00557

**Published:** 2024-08-05

**Authors:** Sumbul Hafeez, Aysha Khanam, Han Cao, Brian P. Chaplin, Wenqing Xu

**Affiliations:** † Department of Civil and Environmental Engineering, Villanova University, Villanova, Pennsylvania 19085, United States; ‡ Department of Chemical Engineering, University of Illinois at Chicago, 929 W. Taylor St., 14, Chicago, Illinois 60607, United States

**Keywords:** per- and polyfluoroalkyl substances (PFAS), perfluorooctanoic acid (PFOA), molecularly imprinted polymer (MIP), electrochemical sensor, conductive polymer, redox-active polymer

## Abstract

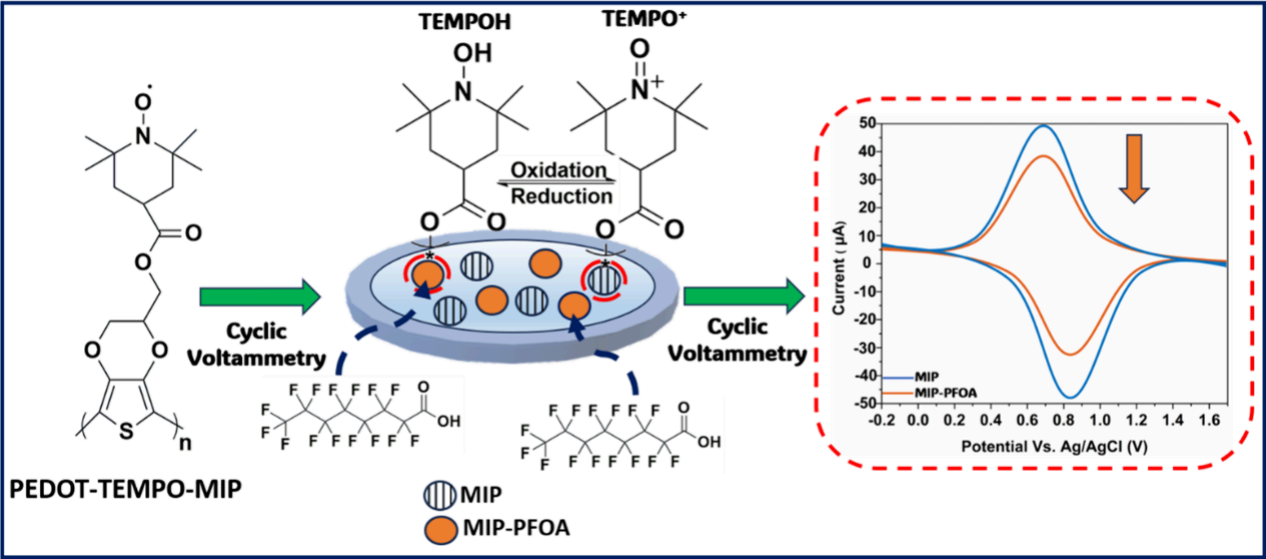

This study developed a novel molecularly imprinted polymer (MIP) that is both conductive and redox-active for directly quantifying perfluorooctanoic acid (PFOA) electrochemically. We synthesized the monomer 3,4-ethylenedioxythiophene-2,2,6,6-tetramethylpiperidinyloxy (EDOT-TEMPO) for electropolymerization on a glassy carbon electrode using PFOA as a template, which was abbreviated as PEDOT-TEMPO-MIP. The redox-active MIP eliminated the need for external redox probes. When exposed to PFOA, both anodic and cathodic peaks of MIP showed a decreased current density. This observation can be explained by the formation of a charge-assisted hydrogen bond between the anionic PFOA and MIP’s redox-active moieties (TEMPO) that hinder the conversion between the oxidized and reduced forms of TEMPO. The extent of the current density decrease showed excellent linearity with PFOA concentrations, with a method detection limit of 0.28 ng·L^–1^. PEDOT-TEMPO-MIP also exhibited high selectivity toward PFOA against other per- and polyfluoroalkyl substances (PFAS) at environmentally relevant concentrations. Our results suggest electropolymerization of MIPs was highly reproducible, with a relative standard deviation of 5.1% among three separate MIP electrodes. PEDOT-TEMPO-MIP can also be repeatedly used with good stability and reproducibility for PFOA detection. This study provides an innovative platform for rapid PFAS quantification using redox-active MIPs, laying the groundwork for developing compact PFAS sensors.

## Introduction

Per- and polyfluoroalkyl substances (PFAS) are a suite of ionizable synthetic organofluorine surfactants widely used in industrial and consumer applications over the past few decades.^1−3^ Some PFAS are highly recalcitrant, bioaccumulative, and toxic.^4,5^ The U.S. Environmental Protection Agency has set maximum contaminant levels (MCLs) for six PFAS in drinking water, with the MCLs for perfluorooctanesulfonic acid (PFOS) and perfluorooctanoic acid (PFOA) at 4 ng·L^–1^ each as of April 2024.^6^ Reports suggest that PFAS are present in the drinking water of 200 million Americans.^7,8^ There is consensus that technologies for rapid PFAS detection at sub ng·L^–1^ concentrations are critical but lacking.

The current gold standard of PFAS detection relies on expensive instrumentation (i.e., liquid chromatography-triple quadrupole-tandem mass spectrometry (LC-MS/MS)), which requires specialized operator training and labor-intensive sample processing that takes hours to days, and is cost-prohibitive.^9−12^ Consequently, an array of optical and electrochemical PFAS sensors have been developed, but their selectivity remains problematic.^13−16^ This aspect is particularly important given that many PFAS exist in mixtures and interfering ions often coexist. Molecularly imprinted polymers (MIPs) offer high selectivity for PFAS due to the molecular “lock-and-key” binding mechanism, which was previously employed for concentrating PFAS from water samples.^17−20^ Briefly, MIPs are cross-linked polymers synthesized in the presence of template molecules (e.g., PFAS), which are removed by post-polymerization to create cavities with a size, shape, and binding affinity similar to the template used.^21−23^ Combining MIPs with electrochemical detection has gained immense attention, which relies on changes in electrochemical signals upon binding and removal of templates from MIP cavities. MIPs are increasingly used in chemical sensing platforms to detect drug residues,^24,25^ organic dyes,^26,27^ pesticides,^28,29^ pathogens,^21,30^ and PFAS.^31−35^ However, several major obstacles exist. First, existing MIPs coupled with electrochemical detection are not redox-active and thus require addition and periodic replenishment of external redox probes (e.g., ferric/ferrocyanide), adding complexity to integration and downsizing of sensor systems.^31−33^ Moreover, current studies employed non-conductive MIPs (e.g., *o-*phenylenediamine),^31−33,36^ limiting electron transfer and signal transduction during measurement. Additionally, several existing conductive MIPs (e.g., polypyrrole and polyaniline) are not sufficiently stable in water, leading to decreased conductivity over time.^37,38^

**Scheme 1:**
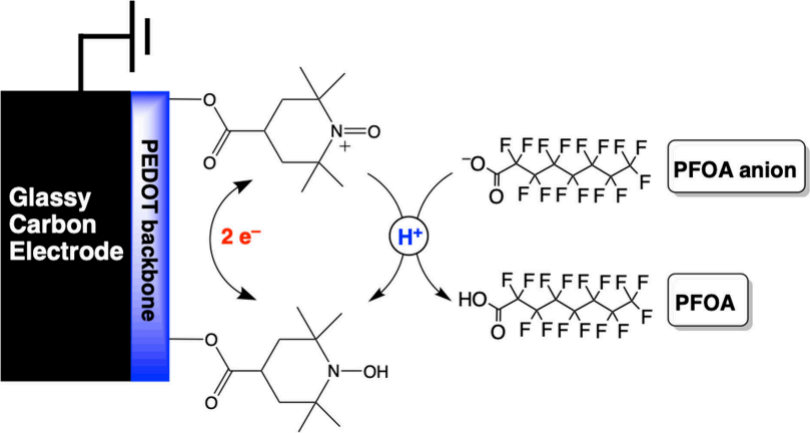
Proposed Mechanism for Electrochemical Signal Decrease upon the Interaction between PFOA Anion and Redox-Active Moieties on PEDOT-TEMPO-MIP (i.e., TEMPO: TEMPOH/TEMPO^+^) that Results in Blockage of Conversion between the Oxidized and Reduced Forms of TEMPO

Herein, we leveraged the high conductivity and robustness of poly-3,4-ethylenedioxythiophene (PEDOT) in water and incorporated 2,2,6,6-tetramethylpiperidinyoxy (TEMPO), a redox-active *N*-oxyl derivative, into PEDOT for direct quantification of PFAS at sub ng·L^–1^ concentrations.^39−43^ The detection mechanism for PFOA relies on proton blocking, hindering the conversion of TEMPOH to TEMPO^+^ upon oxidation and reduction (Scheme 1). Specifically, we synthesized and purified the monomer EDOT-TEMPO, performed electropolymerization to obtain PEDOT-TEMPO-MIP using PFOA as a template, established a calibration curve, and assessed the selectivity, stability, and reusability of PEDOT-TEMPO-MIP towards PFOA in the range that is typical in surface water. Our findings provide an innovative platform for rapid ex-situ PFAS quantification by creating a redox-active MIP, eliminating the need for external redox probes, and laying the groundwork for compact PFAS sensor development.

## Methods and Materials

### Chemicals, Material Characterization, and Chemical Analysis

All chemicals, material characterization, and chemical analysis are provided in the Supporting Information (SI) (Texts S1, S2, and S3).

### Preparation of MIPs

Details on the preparation of monomer EDOT-TEMPO, PEDOT-TEMPO-MIP, and non-molecularly imprinted PEDOT-TEMPO (PEDOT-TEMPO-NIP) are provided in Text S4.^44,45^

### Electrochemical Measurement

All potentials were reported relative to the Ag/AgCl reference electrode. The cyclic voltammetry (CV) scans of glassy carbon electrode, PEDOT-TEMPO-MIP and PEDOT-TEMPO-NIP were collected at a potential range of 0.0–1.5 V with a scan rate of 20 mV·s^–1^ in dichloromethane (DCM) solution containing 0.1 mol·L^–1^ tetrabutylammonium hexafluoro phosphate (TBAPF_6_) as a commonly used nonaqueous electrolyte. DCM was selected due to its high compatibility with PFOA and TBAPF_6_ and was used for MIP electropolymerization and electrochemical signal monitoring. By contrast, the rebinding of PFAS was carried out in DI or actual water samples. The direct quantification of PFOA using PEDOT-TEMPO-MIP involves (1) MIP synthesis by electropolymerization in DCM containing TBAPF_6_, (2) removal of PFOA template with DI, (3) rebinding of PFOA with MIP in DI (or actual water sample), and (4) detection of PFOA captured by MIP in an electrochemical cell containing DCM and TBAPF_6_. Steps (1) and (4) were performed in an electrochemical cell, and steps (2) and (3) were carried out in batch reactors with water. Furthermore, the reference current density (*i*_0_) was recorded in CV scans in an electrochemical cell in DCM for PEDOT-TEMPO-MIP after template (PFOA) removal. Rebinding of PFOA was conducted by exposing PEDOT-TEMPO-MIP to different PFOA concentrations (i.e., 4.14 × 10^–10^ to 4.14 × 10^–4^ g·L^–1^) in DI water. Afterward, PEDOT-TEMPO-MIP was transferred to an electrochemical cell for CV scans in DCM to investigate the impact of PFOA rebinding on the electrochemical signal. The baseline current density (*i*_0_) of PEDOT-TEMPO-MIP was taken at an anodic peak of TEMPO after PFOA template removal, whereas *i* was recorded after being exposed to PFOA in DI water (also referred to as “rebinding process”). Three cycles of CV scans were conducted to obtain a stable electrode response (Figure S1). The average value from the second and third scans was used for each measurement. The changes in current density (i.e., Δ*i* = *i – i*_0_) were plotted against PFOA concentrations for the calibration curve. Each PFOA concentration was measured in triplicate.

## Result and Discussion

### Characterization of EDOT-TEMPO monomer

The successful synthesis of the EDOT-TEMPO monomer was indicated by its orange crystal (Figure S2) and by ^1^H nuclear magnetic resonance (NMR) and Fourier transform infrared (FTIR) spectroscopy (Figures S3 and S4). Text S6 explains the NMR and FTIR spectroscopy in detail.

### Preparation of PEDOT-TEMPO-MIP and PEDOT-TEMPO-NIP

The CV scans were collected during the electropolymerization of PEDOT-TEMPO-MIP with PFOA (Figure S5). Consistent with the literature,^44,45^ we observed a slight shift in the anodic peak of EDOT from 1.2 to 1.4 V and from 0.86 to 0.90 V for TEMPO over ten scans, which was attributed to increased resistivity between the working electrode and reference electrode following the growth of PEDOT-TEMPO-MIP film.^44,46^ The increased current density following electropolymerization can be explained by increased pseudocapacitance of the film by incorporating more redox-active moieties into the MIP.^44,47^ The glassy carbon electrode coated with the PEDOT-TEMPO-MIP shows an apparent blue color (Figure S5).^44,45^

**Figure 1:**
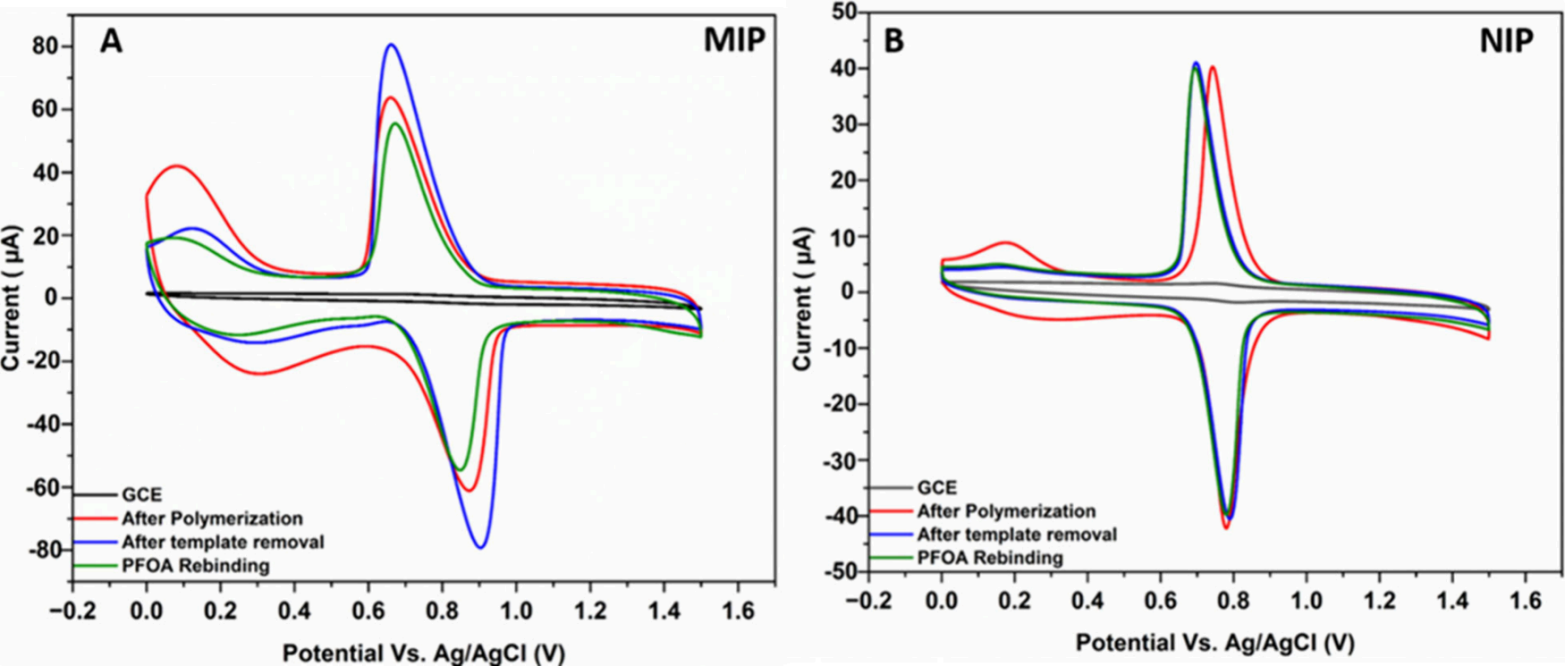
CV scans of bare glassy carbon electrode (black), after electropolymerization (red), after template removal (blue), and after exposure to 4.14 × 10^–4^ g·L^–1^ PFOA for 30 min (green): (A) PEDOT-TEMPO-MIP and (B) PEDOT-TEMPO-NIP.

Two characteristic peaks were observed in CV scans of PEDOT-TEMPO-MIP (Figure 1A; red curve): the anodic and cathodic peaks appeared at 0.86 and 0.69 V, respectively, corresponding to the redox-active TEMPO (i.e., TEMPO^+^, and TEMPOH, Scheme 1).^41−43^ The peaks at 0.30 and 0.18 V suggested anodic and cathodic peaks of the PEDOT backbone.^44,45^ After electropolymerization, PEDOT-TEMPO-MIP was washed with DI water instead of organic solvent to remove the PFOA template. The rationale of solvent selection was explained in Text S4.^17−19^ We observed an increase of 21.1% and 18.5% in current density at anodic and cathodic peaks of PEDOT-TEMPO-MIP, respectively (Figure 1A; blue vs. red curves). When PEDOT-TEMPO-MIP was exposed to 4.14 × 10^–4^ g·L^–1^ PFOA in DI for 30 min during rebinding, a decrease of 40% and 34% in anodic and cathodic peaks current density was observed, respectively (Figure 1A; green curve). We postulate that the observed decrease in the current density in the presence of PFOA can be attributed to the interaction between the PFOA and TEMPO moieties. Specifically, the anionic PFOA (p*K*_a_ = 0.5–3.8)^48,49^ may form a charge-assisted hydrogen bond with TEMPOH (p*K*_a_ = 5.5–6.2). As a result, the conversion from TEMPOH to TEMPO^+^ upon oxidation is hindered, causing a decrease in anodic and cathodic peaks’ current density (Scheme 1). The formation of a charge-assisted hydrogen bond has been previously reported to form between PFOA and polar groups of various surfaces.^50−52^ The closer proximity of p*K*_a_ values of two species, the stronger the H-bond formed.^50−52^

PEDOT-TEMPO-NIP was prepared under the same experimental conditions as PEDOT-TEMPO-MIP, but without PFOA. The CV scan for PEDOT-TEMPO-NIP showed CV characteristics similar to those of PEDOT-TEMPO-MIP (Figure 1B; red curve). However, the current density after removing the PFOA template (Figure 1B; blue curve) or rebinding with PFOA for 30 min (Figure 1B; green curve) exhibited negligible changes at both anodic and cathodic peaks of TEMPO, suggesting a lack of PFOA molecular imprinting cavities in PEDOT-TEMPO-NIP.

### Electrochemical Measurements of PFOA and Construction of Calibration Curve

To determine the optimal rebinding time for PFOA quantification, we first performed time-dependent rebinding experiments by exposing PEDOT-TEMPO-MIP to four different PFOA concentrations (i.e., 4.14 × 10^–10^, 4.14 × 10^–9^ g·L^–1^, 4.14 × 10^–8^ g·L^–1^, and 4.14 × 10^–4^ g·L^–1^). At a higher PFOA concentration (i.e., 4.14 × 10^–4^ g·L^–1^), we observed a decrease in current density by 37% to 85% from 30 to 180 min, which plateaued around 240 min (Table S1). The current density decreased to a lesser extent at a lower PFOA concentration (i.e., 4.14 × 10^–8^ g·L^–1^, 4.14 × 10^–9^ g·L^–1^, 4.14 × 10^–10^ g·L^–1^) compared to a higher PFOA concentration (i.e., 4.14 × 10^–4^ g·L^–1^), and current density plateaued after 210 min. For consistency, we chose 240 min for all subsequent experiments unless otherwise stated.

**Figure 2:**
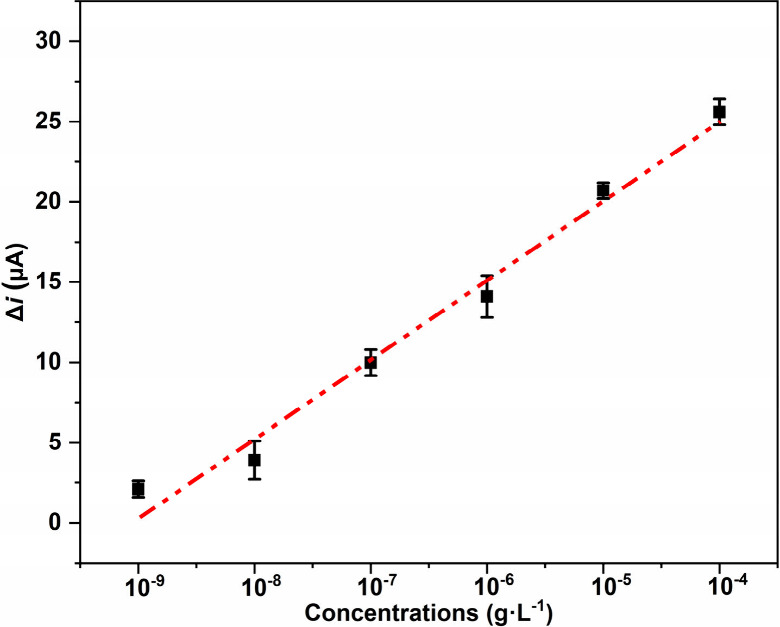
Calibration curve of the current density decrease at the anodic peak of TEMPO (*y*-axis) vs PFOA concentrations (ranging from 4.14 × 10^–9^ to 4.14 × 10^–4^ g·L^–1^; *x*-axis). The current density was recorded from the CV scans of PEDOT-TEMPO-MIP after being exposed to PFOA for 5 min. The error bar at each point was derived from triplicate measurements. The linear regression is *y* = 4.91*x* – 4.47 (*R*^2^ = 0.98).

The calibration curve of PFOA was constructed by obtaining *Δi* from the anodic peak of TEMPO after rebinding with PFOA for 5 and 240 min, respectively, at a series of concentrations (i.e., 4.14 × 10^–10^ g·L^–1^ to 4.14 × 10^–4^ g·L^–1^). The rebinding time of 5 min was selected to establish a more realistic scenario for rapid sensing, as shown in Figure 2, Figure S6, and Table S2. The regression analysis indicated a good linear relationship at both rebinding times (5 and 240 min) (i.e., *R*^2^ = 0.98) between *Δi* and corresponding PFOA concentrations (Figure 2 and Figure S6). A method detection limit (MDL) of 3.26 × 10^–9^ g·L^–1^ for PEDOT-TEMPO-MIP was determined for 5 min exposure of PFOA using seven replicates of the lowest calibration standard (i.e., 4.14 × 10^–9^ g·L^–1^) (Figure 2) following an EPA standard method (Text S5).^53,54^ By contrast, a lower MDL of 2.80 × 10^–10^ g·L^–1^ was achieved for PEDOT-TEMPO-MIP using seven replicates of lowest calibration standard (i.e., 4.14 × 10^–10^ g·L^–1^) for 240 min rebinding (Figure S6; Text S5).^53,54^ Our results suggest that as PFOA concentrations increase, more PFOA molecules were able to block proton transfer to TEMPO^+^ moieties, thereby hindering conversion between TEMPO^+^/TEMPOH and lowering TEMPO anodic peak current density.

### Reproducibility, Stability, and Selectivity of PEDOT-TEMPO-MIP

The reusability of PEDOT-TEMPO-MIP was assessed by evaluating the relative change in current density of the anodic peak of TEMPO at 0.87 V during five successive measurements of the same PFOA sample (Figure S7). The relative standard deviation (RSD) of five measurements was 6.5%. Although the reference current density (*i*_0_) shifted slightly from −125 to −117 μA, possibly due to some irreversible bindings of PFOA on MIPs, the current density (*i*) shifted accordingly through consecutive measurements (i.e., from −116 to −108 μA). Our results suggest that PEDOT-TEMPO-MIP can be reused at least five times with good stability and reproducibility. Furthermore, the reproducibility of PEDOT-TEMPO-MIP was demonstrated by measuring 4.14 × 10^–9^ g L^–1^ PFOA using three different electrodes. The change in current density of the anodic peak of TEMPO at 0.87 V after washing and rebinding with 4.14 × 10^–9^ g L^–1^ PFOA was observed for each MIP electrode, and values are summarized in Table S3. Our results suggest that electropolymerization of MIPs was highly reproducible with a relative standard deviation (RSD) of 5.1%.

The selectivity of PEDOT-TEMPO-MIP was evaluated by monitoring the current density decrease of anodic peak of TEMPO at 0.87 V when exposed to (i) a single PFOA solution (4.14 × 10^–8^ g·L^–1^), (ii) a single non-PFOA solution, namely, perfluorobutanoic acid (PFBA), PFOS, or 6:2 fluorotelomer sulfonamide alkylbetaine (6:2 FTAB) solution (4.14 × 10^–8^ g·L^–1^), and (iii) a mixture of PFOA/PFBA, PFOA/PFOS, or PFOA/6:2 FTAB solution (each at 4.14 × 10^–8^ g·L^–1^). The current density decreased by 18.4 ± 1.1% when PEDOT-TEMPO-MIP was exposed to single PFOA solution (i.e., 4.14 × 10^–8^ g·L^–1^). By contrast, the current density was only reduced by 2.7 ± 0.8%, 1.4 ± 0.6%, and 2.5 ± 0.6% in the presence of a non-template molecule, such as PFBA, PFOS, or 6:2 FTAB solution (i.e., 4.14 × 10^–8^ g·L^–1^), respectively, suggesting that none of these compounds was able to effectively block proton transfer and hinder subsequent conversion between TEMPOH and TEMPO^+^. PEDOT-TEMPO-MIP showed high selectivity toward PFOA against its structural analogs (i.e., PFBA, PFOS, and 6:2 FTAB), which can be explained by the formation of the exact shape and size cavity of template molecule (PFOA) as compared to a non-template molecule with different chain length, headgroup, and charge.^17−19^ When PEDOT-TEMPO-MIP was exposed to a mixture of PFOA/PFBA, PFOA/PFOS, and PFOA/6:2 FTAB (each at 4.14 × 10^–8^ g·L^–1^), current density decreased by 17.2 ± 3.0%, 14.6 ± 2.8%, and 20.2 ± 3.2%, respectively. Our results from the PFOA mixture system were not significantly different from those of a single PFOA in DI, highlighting the selectivity of MIPs toward PFOA in the presence of its structural analogs.

**Figure 3:**
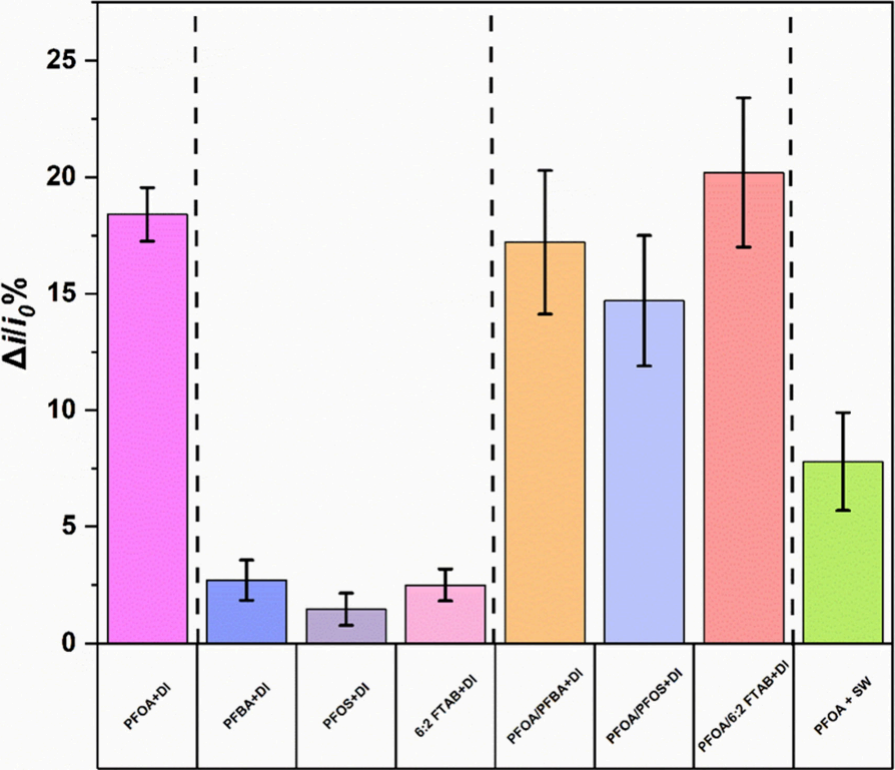
Decrease in current density at the anodic peak of TEMPO of PEDOT-TEMPO-MIP after rebinding with (i) a single PFOA, PFBA, PFOS or 6:2 FTAB (each at 4.14 × 10^–8^ g·L^–1^) in DI water, (ii) a mixture of PFOA/PFBA, PFOA/PFOS, or PFOA/6:2 FTAB (each at 4.14 × 10^–8^ g·L^–1^) in DI water, and (iii) spiked a known concentration of PFOA (4.14 × 10^–8^ g·L^–1^) into the surface water sample for 240 min.

Lastly, we evaluated the performance of PEDOT-TEMPO-MIP in a surface water sample. The chemical composition and sampling location are provided in Table S4 and Figure S8. Specifically, surface water contains a 7.3 ± 0.4 mg·L^–1^ non-purgeable organic carbon (NPOC) and 7.8 ± 0.1 mg·L^–1^ chloride anion. The absence of PFOA and other PFAS such as PFOS, PFBS, perfluorohexanoic acid (PFHxA), hexafluoropropylene oxide dimer acid (HFPO-DA), and 6:2-fluorotelomersulfonic acid (6:2-FTS) in DI water and collected surface water sample was confirmed by LC-MS/MS. To understand the impact of water matrices on PFOA quantification using PEDOT-TEMPO-MIP, we spiked a known concentration of PFOA (4.14 × 10^–8^ g·L^–1^) into the surface water sample. The relative current density change of the anionic peak of TEMPO after rebinding was monitored following the same protocol. We observed a decrease in the current density of MIP (Figure 3) by 7.8 ± 2.1% in a surface water sample, which was 18.4 ± 1.1% lower than when MIP was exposed to the same concentration of PFOA in DI water. The decrease in current density in surface water compared to DI can be attributed to the presence of interfering ions (Cl^–^, SO_4_^2–^) that are small enough to occupy MIP cavities but could not hinder H^+^ transfer between TEMPOH and TEMPO^+^ due to the lack of ability to form H-bond.

## Environmental Significance

This work demonstrates the feasibility of synthesizing PEDOT-TEMPO-MIP that can directly quantify sub ng·L^–1^ PFAS concentrations electrochemically without using external redox probes. Specifically, we directly quantify PFOA with MIP by utilizing specific interactions between PFOA and binding sites that cause signal reduction. By contrast, past published methods rely on external redox probes, which quantify PFOA indirectly and are more likely to result in a false positive. Our obtained MDL for PEDOT-TEMPO-MIP is significantly lower than reported MDL values from potentiometric, fluorescence, and calorimetric sensors for PFAS (10^–5^ to 10^–3^ g·L^–1^)^55−57^ and slightly lower than reported MDLs for photoelectrochemical, electrochemical impedance spectroscopy, and Raman spectroscopy sensors (10^–9^ to 10^–8^ g·L^–1^).^58−60^ It is worth noting that our calculated MDL of PEDOT-TEMPO-MIP may be further improved by applying other electrochemical techniques (e.g., differential pulse voltammetry or square wave voltammetry) to reduce background noise and enhance sensitivity.^31,47^

Moreover, a novel PFAS sensing mechanism was elucidated for the first time. Specifically, we utilized redox-active properties of PEDOT-TEMPO-MIP, namely, the conversion between TEMPOH and TEMPO^+^ upon oxidation and subsequent reduction, to directly quantify PFAS. The aspect of directly quantifying PFAS is of significance for environmental monitoring. Existing electrochemical detection of PFAS relies on external redox probes (e.g., ferric/ferrocyanide), which must be added and replenished periodically.^31−33^ The ability to directly quantify PFAS helps simplify component integration for sensor platforms and enables the development of portable devices. The reversible nature of the redox-active TEMPO also helps eliminate the need for reagent replenishment and lays the groundwork for continuous monitoring, which may revolutionize the field of environmental monitoring.

Lastly, the ability to rapidly quantify PFAS is highly desirable in the field. For instance, current gold-standard LC-MS/MS requires complex sample preparation and analysis protocols, which take hours to days to obtain results.^3,10,12,59^ The typical sample preparation and testing time for surface-enhanced raman spectroscopy (SERS) is in the range of hours, which relies on a diffusion-controlled process to accumulate sufficient PFAS between neighboring nanoparticles to reach the best signal enhancement.^61−63^ By contrast, PFAS detection in our proposed platform can be accomplished within a few minutes (e.g., 5 min), which lays the groundwork for rapid detection, that is urgently needed in environmental science and engineering. Although some loss of sensitivity was observed due to interfering ions in water matrices, presumably, the calibration curve can be prepared in the same water matrices but at higher concentrations than native water, which might help correct background interference. Nonetheless, our PFAS sensor can serve as a screening tool to quickly identify water bodies impacted by PFAS pollution, allowing further PFAS characterization. Future work on the scale-up feasibility of MIP-coupled electrochemical sensors, cost analysis, and potential for real-time sensing are warranted.

## Supporting Information Available

Details of chemical and reagents (Text S1); material characterization (Text S2); chemical analysis (Text S3); preparation of MIPs (Text S4); synthesis and purification of 3,4-ethylenedioxythiophene-2,2,6,6-tetramethylpiperdinyloxy (EDOT-TEMPO) monomer, electrochemical polymerization of poly-3,4-ethylenedioxythiophene-2,2,6,6-tetramethylpiperdinyloxy (PEDOT-TEMPO-MIP) and nonmolecularly imprinted polymers (PEDOT-TEMPO-NIP); method detection limit (MDL) calculation (Text S5); characterization of EDOT-TEMPO monomer (Text S6); scheme for the synthesis of EDOT-TEMPO monomer via the esterification reaction between EDOT-MeOH and TEMPO-COOH at room temperature for 72 h (Scheme S1); scheme for the synthesis of PEDOT-TEMPO-MIP using 1 mmol·L^–1^ EDOT-TEMPO and 1 mmol·L^–1^ PFOA in a 0.1 M TBAPF_6_ dichloromethane solution, employing a scan rate of 20 mV·s^–1^ and a potential range of 0–1.5 V. (Scheme S2); three cycles of cyclic voltammograms (CV) scans of PEDOT-TEMPO-MIP after template removal in 0.1 M TBAPF_6_ dichloromethane solution (Figure S1); orange color crystal of EDOT-TEMPO monomer after purification (Figure S2); ^1^H NMR spectrum of EDOT-TEMPO (Figure S3); FTIR spectrum of EDOT-TEMPO (Figure S4); ten cycles of CV scans during electropolymerization of PEDOT-TEMPO-MIP using EDOT-TEMPO in 0.1 M TBAPF_6_ dichloromethane solution (Figure S5); the calibration curve of the current density decreases at the anodic peak of TEMPO (*y*-axis) vs PFOA concentrations ranging from 4.14 × 10^–9^ to 4.14 × 10^–4^ g·L^–1^ (*x*-axis). (Figure S6); the relative changes in the current density of TEMPO’s anodic peak at 0.87 V over 5 cycles of washing (template removal) and rebinding (when exposed to 4.14 × 10^–9^ g·L^–1^ PFOA). (Figure S7); the sampling location of the surface water from a pond near West Windsor Township, New Jersey, USA (Figure S8); the decrease in current density of the anodic peak of TEMPO during PFOA rebinding at different time intervals (Table S1); the decrease in current density of the anodic peak of TEMPO after being exposed to PFOA in a concentration range of 4.14 × 10^–4^ g·L^–1^ to 4.14 × 10^–9^ g·L^–1^ for 5 min (Table S2); reproducibility of PFOA (4.14 × 10^–9^ g L^–1^) measurements using three PEDOT-TEMPO-MIP electrodes (Table S3) and the chemical composition of the collected surface water sample (Table S4) (PDF)
