# Correction to “Novel Conductive and Redox-Active Molecularly Imprinted Polymer for Direct Quantification of Perfluorooctanoic Acid”

**DOI:** 10.1021/acs.estlett.4c01158

**Published:** 2025-01-10

**Authors:** Sumbul Hafeez, Aysha Khanam, Han Cao, Brian P. Chaplin, Wenqing Xu

In our recently published work, we discovered an error in Figure 1 in the original manuscript. The error is related to plotting the cyclic voltammetry (CV) scans. Furthermore, Figure 1 has been corrected according to the IUPAC convention in which the anodic current should be positive (up) and the cathodic current should be negative (down). The revised version of Figure 1 is below. The correction does not impact any of the conclusions discussed in the manuscript.

**Figure 1:**
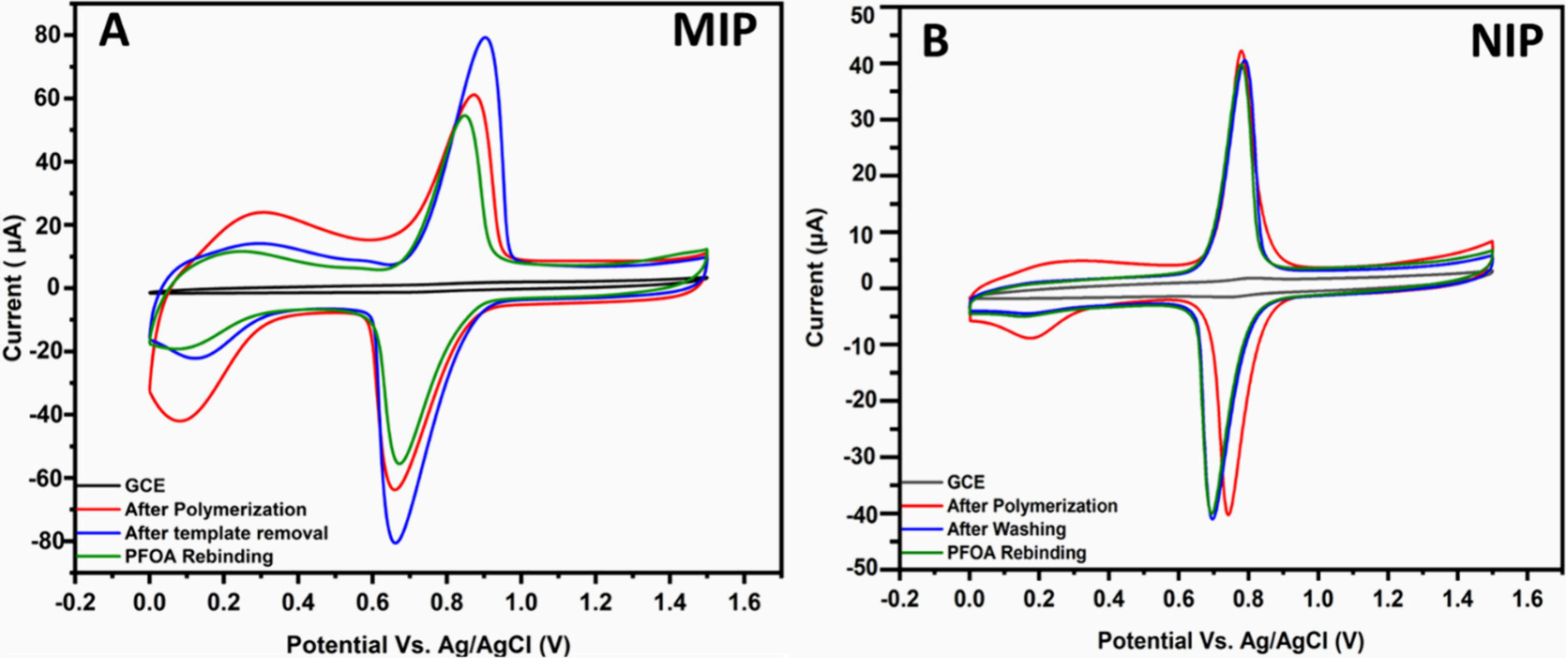
CV scans of bare glassy carbon electrode (black), after electropolymerization (red), after template removal (blue), and after exposure to 4.14 × 10^–4^ g·L^–1^ PFOA for 30 min (green): (A) PEDOT-TEMPO-MIP and (B) PEDOT-TEMPO-NIP.

