# Supplementary material for: Novel Conductive and Redox-Active Molecularly Imprinted Polymer for Direct Quantification of Perfluorooctanoic Acid
Source: Environ Sci Technol Lett. 2024 Aug 5;11(8):871–7. doi: 10.1021/acs.estlett.4c00557 (PMC11325644; doi:10.1021/acs.estlett.4c00557)
Supplement: Supplementary file 1 — ez4c00557_si_001.pdf [file ez4c00557_si_001.pdf]

SUPPORTING INFORMATION

Novel Conductive and Redox-Active Molecularly Imprinted Polymer for Direct Quantification of Perfluorooctanoic Acid

Sumbul Hafeez[1], Aysha Khanam[1], Han Cao[1], Chaplin Brian P.[2], Wenqing Xu[1, *]

[1]Department of Civil and Environmental Engineering, Villanova University, Villanova, Pennsylvania 19085, United States

[2]Department of Chemical Engineering, University of Illinois at Chicago, 929 W. Taylor St., 14 Chicago, IL 60607, United States

[*]Corresponding author:

Dr. Wenqing Xu

Email: wenqing.xu@villanova.edu

Phone : 610-519-8549

16 pages

4 tables

8 figures

6 textes

Text S1: Chemical and Reagents

3,4-ethylenedioxythiophene hydroxymethyl (EDOT-MeOH, ≥95%), 4-carboxy-2,2,6,6-tetra methylpiperidinyloxy **(**TEMPO-COOH, ≥97%), 4-(dimethylamino)pyridine (DMAP, ≥ 99%), *N*-(3-dimethylaminopropyl)-*N′*-ethylcarbodiimide hydrochloride (EDC, ≥97%) and anhydrous dichloromethane (DCM, ≥99%), were obtained from TCI (Tokyo, Japan) and used as received. Sodium chloride (NaCl, ≥99.5%) was purchased from Fisher Scientific (Pittsburgh, PA) and used as received. Perfluorooctanoic acid (PFOA, ≥97%), perfluorobutanoic acid (PFBA, ≥98%), Perfluorooctanesulfonic acid solution (PFOS, ≥97%), dry tetrabutylammonium hexafluorophosphate ($TBAPF_6$, ≥99%), phenylhydrazine (97%), anhydrous magnesium sulfate ($MgSO_4$, ≥99.7%), hexane (99.9%, HPLC Grade), dichloromethane (99.9%, HPLC Grade), and ethyl acetate (99.9%, HPLC Grade) were purchased from Sigma-Aldrich (Milwaukee, MI) and used as received. 6:2 Fluorotelomer sulfonamide alkylbetaine (6:2 FTAB, ≥97%) was obtained from TRC (Connecticut, USA). Suwannee River natural organic matter (SRNOM) was purchased from the International Humic Substances Society (IHSS, MN, USA). Deionized (DI) water was purified by a Mili-Q-plus purification system (≥18.2 MΩ cm at 25 °C; Millipore Sigma, MO, USA). Surface water (SW) was collected from a pond (latitude and longitude of 40.310329 and -74.644513°, respectively) in West Windsor Township, New Jersey. The sampling location and the surface water composition are provided in **Figure S7** and **Table S3.**

Text S2: Material Characterization.

^{1}H nuclear magnetic resonance (NMR) spectra were recorded for EDOT-TEMPO monomer at 25 °C on a JEOL ECZ400S, 400 MHz spectrometer using deuterated chloroform as a solvent and tetramethyl silane as an internal standard. Fourier Transform Infrared Spectroscopy measurements

(FTIR) were recorded on a Perkin Elmer 1600 Series FTIR Spectrophotometer. The spectrum was collected in 32 scans with a resolution of 4 cm^{-1} in the 4000-400 cm^{-1} range.

Text S3: Chemical Analysis

The non-purgeable organic carbon (NPOC) was analyzed with a TOC analyzer (TOC-L, Shimadzu, Japan). The cations (Na^+, K^+, Mg^+, and Ca^+) and anions (Cl^- and SO_4^{2-}) were analyzed with an ion chromatography (IC) coupled with a conductivity detector using a Shodex YS-50 column (for cations) or Shodex SI-52 4E column (for anion) (Showa Denko, Tokyo, Japan), respectively. The turbidity and conductivity of the surface water sample was measured on a turbidity meter (2100Q, HACH) and a conductivity meter (HQ40d, HACH), respectively. PFAS analysis was performed on an Agilent 6470A triple quadrupole LC/MS (LC/QQQ-MS) system C18 column (Agilent poroshell 120 EC, 50 × 3 mm, 1.8 μm) with the mobile phases of solvent A (5 mM ammonium acetate in distilled water) and B (5 mM ammonium acetate in 100% methanol). The oven temperature was set at 40 °C. The injection volume of each sample was 5 µL with a flow rate of 0.5 ml·min^{-1}. Prior to analysis, all samples were filtered by 0.22-µm PES filters with 1-mL syringes into polypropylene 1.5-mL autosampler vials (ThermoFisher) with polypropylene caps. An Agilent 6470A triple quadrupole LC-/MS/MS system was used to detect quantify the concentrations of EPA 537 standards, EPA 533 standards, and intermediates (C2 ~ C7) based on USEPA Method 533 following mass calibration and initial calibration. Analytical sequences will include instrument blanks, instrument sensitivity checks, calibration verification standards, qualitative identification standards, method blanks, ongoing precision and recovery standards (OPRs), and experimental samples. A C18 column (Agilent poroshell 120 EC, 50 × 3 mm, 1.8 μm) was used for separation at 40 °C using a mobile phase of solvent A (5 mM ammonium acetate in distilled water) and B (5 mM ammonium acetate in 100% methanol). The injection volume of each

sample is 5 μL with a flow rate of 0.5 ml·min^{-1}. The EPA 533 standard (500 ppb) (Lot No.537PDSLR11021) purchased from Wellington laboratories were diluted with 100% methanol to, 100 ppt, 250 ppt, 500 ppt, 1 ppb, 2.5 ppb, 5 ppb, 10 ppb, 25 ppb and 50 ppb, respectively, to establish the calibration curve.

Text S4: Preparation of MIPs.

Synthesis and purification of 3,4-ethylenedioxythiophene-2,2,6,6-tetramethyl piperidinyloxy (EDOT-TEMPO) monomer. We prepared the 3,4-ethylenedioxythiophene-2,2,6,6-tetramethylpiperidinyloxy (EDOT-TEMPO) monomer by the esterification reaction between 3,4-ethylenedioxythiophene hydroxymethyl and 4-carboxy-2,2,6,6-tetramethyl piperidinyloxy in the presence of *N*-(3-dimethylaminopropyl)-*N′*-ethylcarbodiimidehydrochloride (EDC) and 4-(dimethylamino) pyridine under dry dichloromethane (DCM) following the **Scheme S1**.[3,4] Briefly, TEMPO-COOH (1.4 mmol, 280 mg,) EDOT-MeOH (2.18 mmol, 380 mg), and DMAP (1.34 mmol, 17 mg) were dissolved in 10 mL of dry DCM in a 100 mL round bottom flask for 5 minutes under stirring at 0 ºC. After 5 min, EDC (1.52 mmol, 310 mg) was dissolved in 4 mL of dry DCM and added dropwise into the round bottom flask at 0ºC under stirring. The reactor was then warmed up to room temperature and stirred for 72 h. After 72 h, the reaction solution was diluted with 20 mL DCM, and the organic phase was washed with NaCl (40 mL) and water (80 mL), then dried over anhydrous $MgSO_4$ and filtered using 11 µm Whatman paper. The filtrate was purified by column chromatography (SiO_2, *n*-hexane/ethyl acetate 1:3 volume ratio) and followed by recrystallization in cold *n*-hexane. The obtained EDOT-TEMPO monomer appeared as orange color crystals, as shown in **Figure S2**. For ^{1}H NMR analysis, phenylhydrazine was added to reduce the nitroxide free radical to its hydroxylamine derivative.

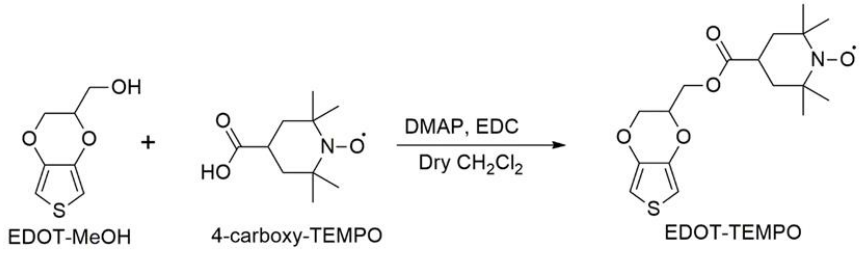

Scheme S1. Scheme for the synthesis of EDOT-TEMPO monomer via the esterification reaction between EDOT-MeOH and TEMPO-COOH at room temperature for 72 h.

Electrochemical polymerization of poly-3,4-ethylenedioxythiophene-2,2,6,6-tetramethylpiperidinyloxy (PEDOT-TEMPO-MIP) and non-molecularly imprinted polymers (PEDOT-TEMPO-NIP). PEDOT-TEMPO-MIP and PEDOT-TEMPO-NIP were prepared by the electrochemical polymerization of EDOT-TEMPO monomer following a method established from previous studies.[1,2] The reaction scheme was shown in **Scheme S2**. The glassy carbon electrode was first polished using 0.3 and 0.05 µm alumina slurry and washed using deionized water for 5 min under a sonication bath. A standard three-electrode configuration was employed with a 3 mm glassy carbon working electrode (BASi, IN, USA), a coiled platinum wire counter electrode (BASi, IN, USA), and an Ag/AgCl reference electrode in 3 M KCl (BASi, IN, USA). All potentials were applied against Ag/AgCl reference electrode in 0.1 mol·L^{-1} $TBAPF_6$ DCM solution. A CH Instruments 630C potentiostat (Austin, TX) was used for electrochemical polymerization and PFOA quantification. Specifically, the electrochemical polymerization of the EDOT-TEMPO (1 mmol·L^{-1}) was performed in 0.1 mol·L^{-1} $TBAPF_6$ in DCM at a scan rate of 20 mV·s^{-1} for 10 sweep cycles on the surface of the glassy electrode under cyclic voltammetry (CV) with a potential range from 0-1.5 V. MIPs were prepared in the presence of 1 mmol·L^{-1} of PFOA, whereas NIP was synthesized under the same condition except in the absence of PFOA. After the completion of the electrochemical polymerization, the electrode was washed with deionized water

to remove the PFOA template. Herein, we have chosen DI water for PFOA template removal because organic solvents such as methanol, ethanol, and acetone can dissolve PEDOT-TEMPO-MIP.[3–5] Moreover, the use of DI for template removal and MIP regeneration is also cost-effective compared to other technologies where organic solvents are often employed.

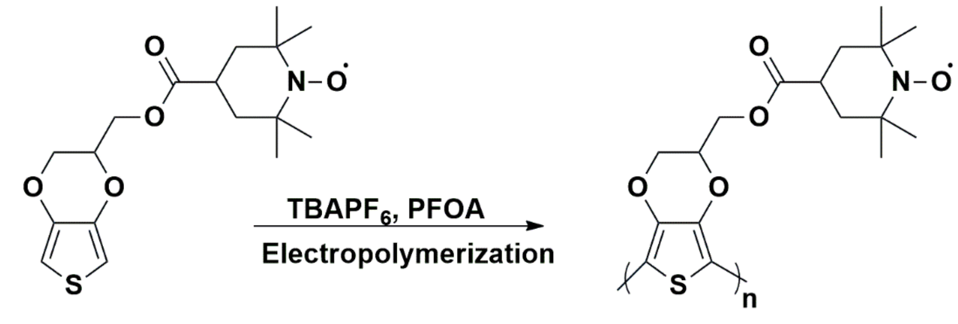

EDOT-TEMPO PEDOT-TEMPO-MIP

Scheme S2. Scheme for the synthesis of PEDOT-TEMPO-MIP using 1 $mmol\cdot L^{-1}$ EDOT-TEMPO and 1 $mmol\cdot L^{-1}$ PFOA in a 0.1 M TBAPF6 dichloromethane solution, employing a scan rate of 20 $mV\cdot s^{-1}$ and a potential range of 0-1.5 V.

Text S5: Method Detection Limit (MDL) calculation

The MDL was calculated using the standard deviation (SD) of seven replicates at the lowest PFOA concentration used for the rebinding experiments (i.e., 4.14×10^{-10} $g\cdot L^{-1}$ and 4.14×10^{-9} $g\cdot L^{-1}$) following an EPA standard method.[6,7] Specifically, the standard deviation of the measured concentrations of seven replicates were determined using the calibration curve (i.e., Figure 2, R^2=0.98), and the MDL was calculated following the equation below:

$$MDL = {}_{(n\text{-}1,1-\propto=0.99)}\cdot SD$$

where MDL represents the method detection limit, $t_{(n-1,1-\alpha=0.99)}$ represents Student's t-value for a single tailed 99th percentile at the degrees of freedom of 6, and SD represents the standard deviation derived from 7 replicates.

Text S6: Characterization of EDOT-TEMPO monomer

The ^{1}H NMR spectrum (**Figure S3**) of EDOT-TEMPO shows two distinctive resonance peaks at 4.6 and 4.2 ppm corresponding to the methylene protons adjacent to carbonyl carbon bonded to the ester oxygen ($-CH_2-COO-$), suggesting the successful esterification of EDOT-MeOH and TEMPO-COOH.[44,45] The resonance at 1.2, 1.4, 1.8, and 2.6 ppm represent the TEMPO-COOH protons, while those at 6.4 and 3.9 ppm correspond to the EDOT-MeOH protons.[44,45] The resonance peaks at 6.8 and 7.15 ppm (crossed out in red) represent the phenylhydrazine protons, which were used to reduce the nitroxide free radical to its hydroxyl derivative.[44,45] Our FTIR results further support these observations (**Figure S4**). Specifically, the vibrational stretching peak shift from 1690 cm^{-1} to 1732 cm^{-1} corresponds to the conversion of carboxylic carbonyl to ester carbonyl. The disappearance of the hydroxyl peak at 3200 cm^{-1} indicates successful esterification of EDOT-MeOH and TEMPO-COOH.[44,45]

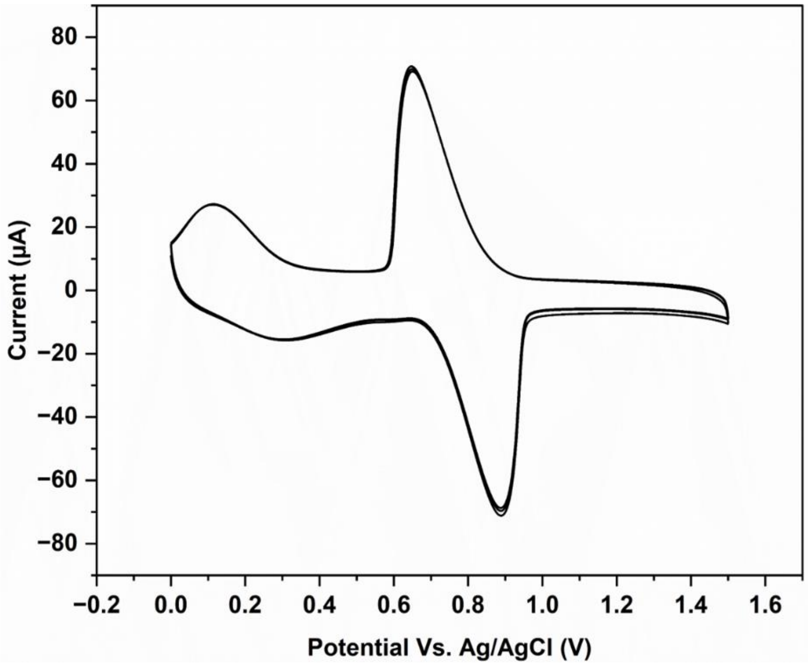

Figure S1. Three cycles of cyclic voltammograms (CV) scans of PEDOT-TEMPO-MIP after template removal in 0.1 M $TBAPF_6$ dichloromethane solution.

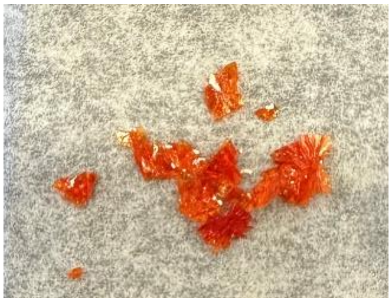

Figure S2. Orange color crystal of EDOT-TEMPO monomer after purification.

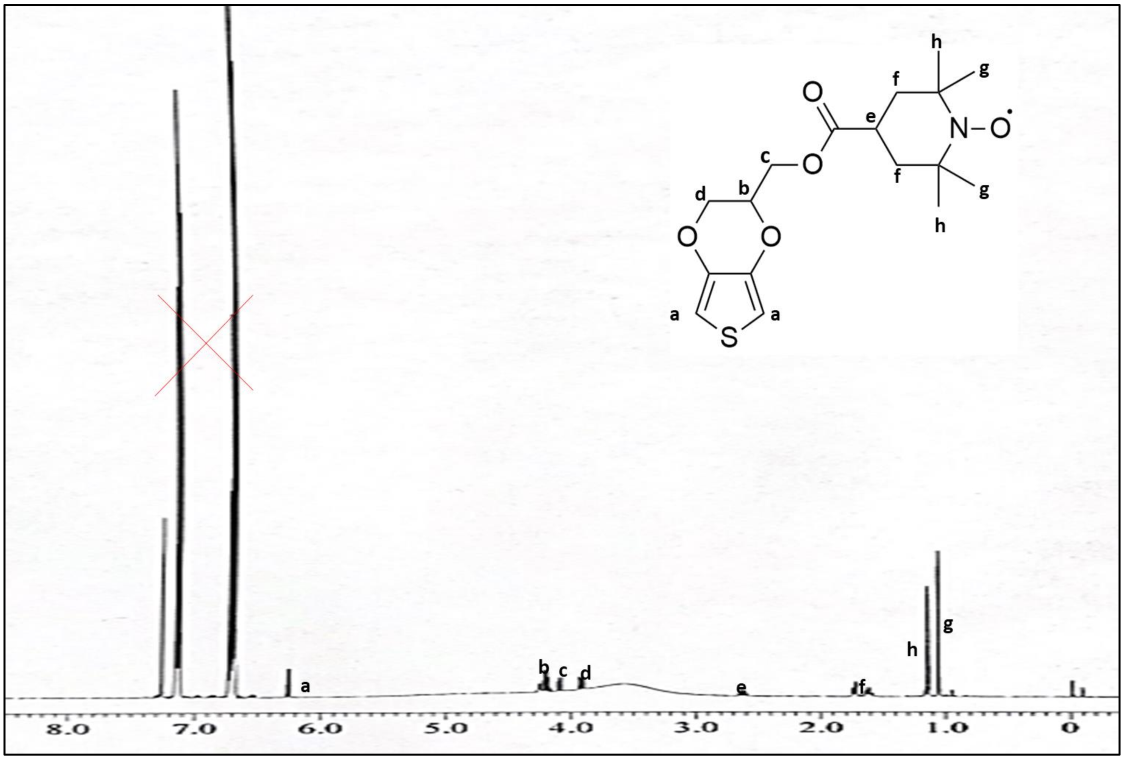

Figure S3. ^{1}H NMR spectrum of EDOT-TEMPO synthesized *via*. the condensation reaction of EDOT-MeOH and TEMPO-COOH at room temperature for 72 h.

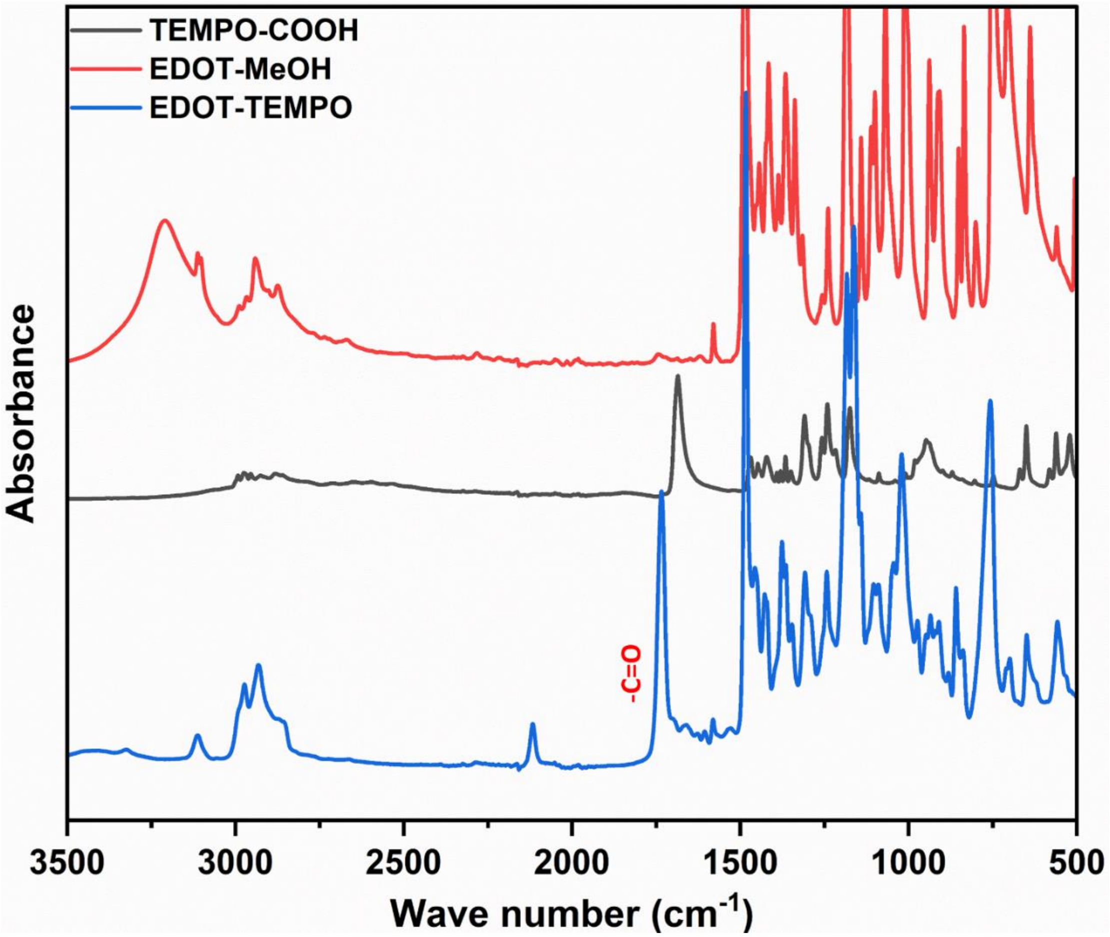

Figure S4. FTIR spectrum of EDOT-TEMPO synthesized *via*. the condensation reaction of EDOT-MeOH and TEMPO-COOH at room temperature for 72 h.

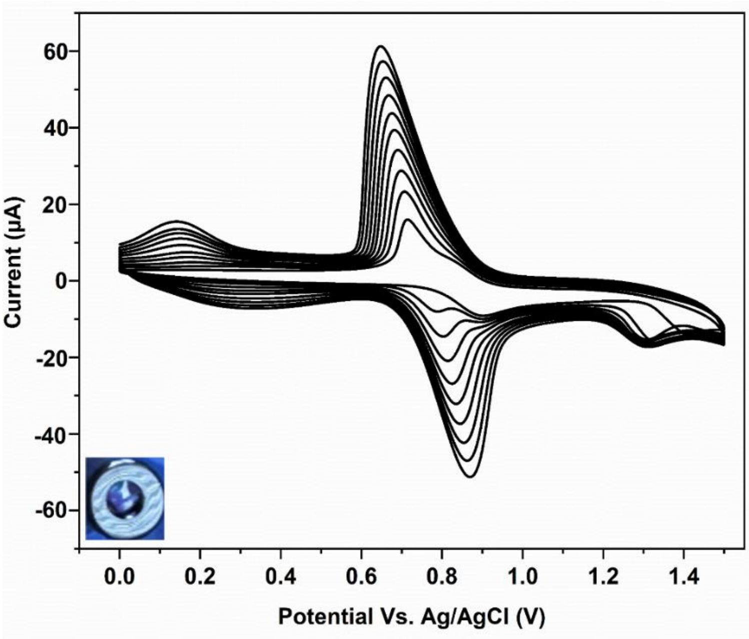

Figure S5. Ten cycles of CV scans during electropolymerization of PEDOT-TEMPO-MIP using EDOT-TEMPO in 0.1 M $TBAPF_6$ dichloromethane solution. The scan rate was at 20 $mV \cdot s^{-1}$ and the potential used range from 0-1.5 V. The inset image shows the blue color film deposited after electropolymerization.

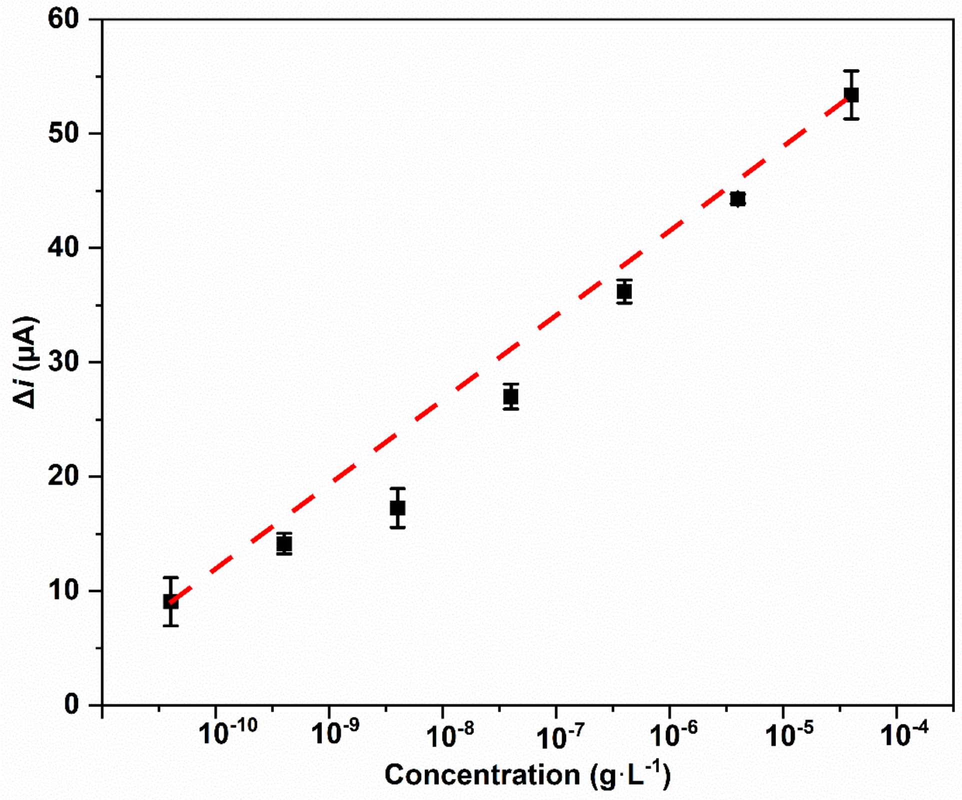

Figure S6. The calibration curve of the current density decreases at the anodic peak of TEMPO (y-axis) vs. PFOA concentrations ranging from 4.14×10^{-10} to 4.14×10^{-4} $g\cdot L^{-1}$ (x-axis). The current density was recorded from the CV scans of PEDOT-TEMPO-MIP after being exposed to PFOA for 240 minutes. The error bar at each point was derived from triplicate measurements. The linear regression is $y = 0.75x - 0.15$ ($R^2 = 0.98$).

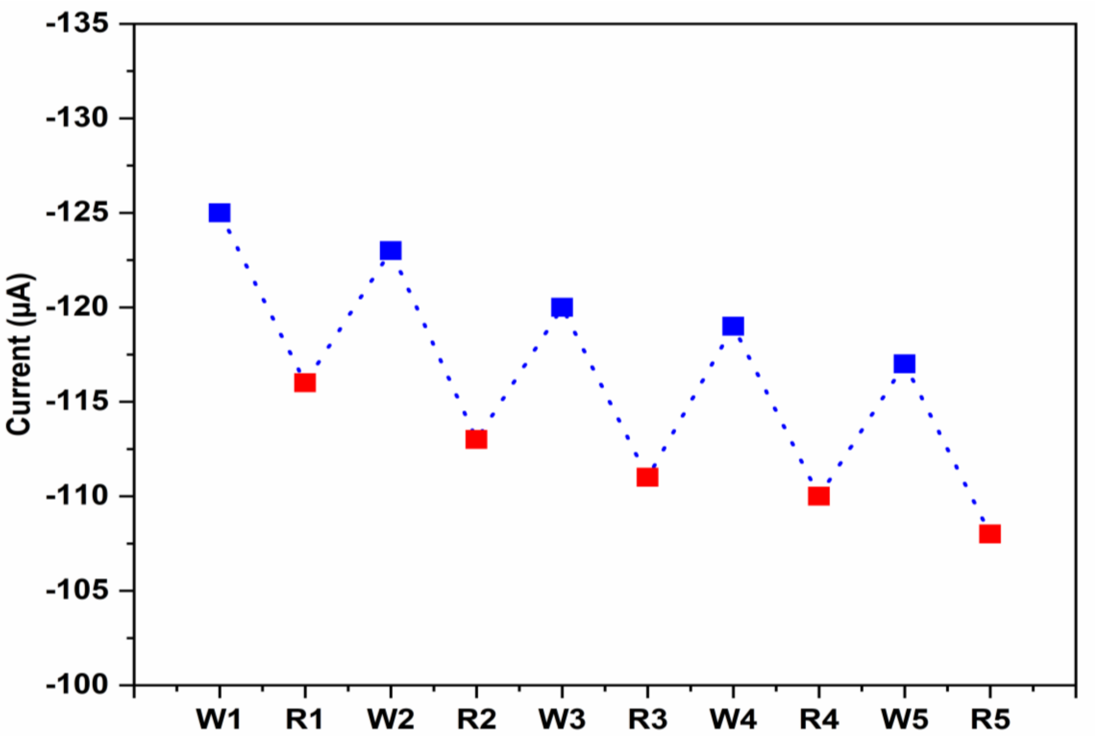

Figure S7. The relative changes in the current density of TEMPO's anodic peak at 0.87 V over 5 cycles of washing (template removal) and rebinding (when exposed to 4.14×10^{-9} $g\cdot L^{-1}$ PFOA). Results from the washing and rebinding cycles are abbreviated as RX and WX, where X represents the number of cycles.

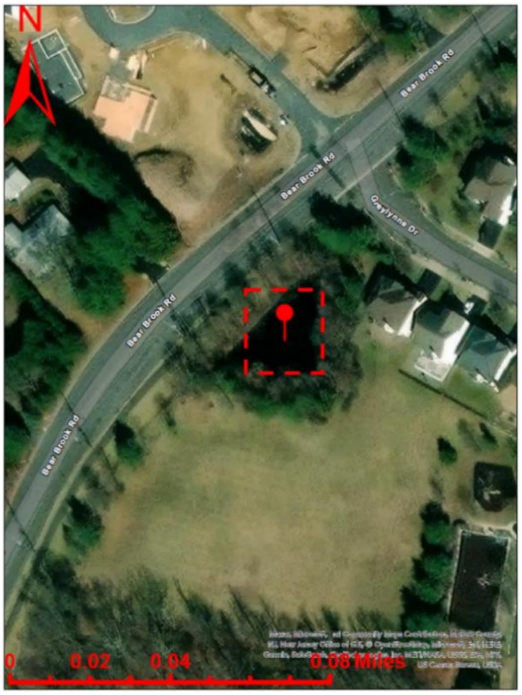

Figure S8. The sampling location of the surface water from a pond near West Windsor Township, New Jersey, USA (40.310329°, -74.644513°).

Table S1. The decrease in current density of the anodic peak of TEMPO during PFOA rebinding at different time intervals.

**Time (minutes)**	**$\Delta i / i_0$% (4.14×10^{-4} g·L^{-1} PFOA)**	**$\Delta i / i_0$% (4.14×10^{-8} g·L^{-1} PFOA)**	**$\Delta i / i_0$% (4.14×10^{-9} g·L^{-1} PFOA)**	**$\Delta i / i_0$% (4.14×10^{-10} g·L^{-1} PFOA)**
2	19.4 %	2.0 %	0 %	0%
5	25.6 %	3.9 %	2.1 %	0%
30	37.3%	15.3 %	7.3 %	0%
60	48.5%	24.8 %	15.8 %	10.0%
90	56.8%	35.4 %	22.2 %	15.0%
120	72.1%	47.6 %	31.6 %	18.5%
150	80.0%	56.4 %	40.4 %	30%

180	85.0%	65.4 %	59.7 %	48.3%
210	86.6%	74.1 %	68.9 %	67.5%
240	87.3%	78.5 %	71.5 %	69.1%

Table S2. The decrease in current density of the anodic peak of TEMPO after being exposed to PFOA in a concentration range of 4.14×10^{-4} g·L^{-1} to 4.14×10^{-9} g·L^{-1} for 5 minutes.

Concentration (g·L^{-1})	$\Delta i = i - i_0$ (μA)
4.14×10^{-4}	25.6 ± 0.8
4.14×10^{-5}	20.7 ± 0.5
4.14×10^{-6}	14.1 ± 1.3
4.14×10^{-7}	9.9 ± 0.8
4.14×10^{-8}	3.9 ± 1.2
4.14×10^{-9}	2.1 ± 0.5

i = current density at the anodic peak of TEMPO after exposed to PFOA for 5 minutes, i_0 = current density at the anodic peak of TEMPO after template removal.

Table S3: Reproducibility of PFOA (4.14×10^{-9} g L^{-1}) measurements using three PEDOT-TEMPO-MIP electrodes

MIP electrode	i	i_0	$\Delta i = i - i_0$ (μA)
1	-118.0	-126.4	8.4
2	-115.0	-124.0	9.0
3	-117.7	-127.0	9.3

i = current density at the anodic peak of TEMPO after exposed to PFOA for 240 minutes, i_0 = current density at the anodic peak of TEMPO after template removal.

Table S4. The chemical composition of the collected surface water sample.

Analyte	Units	Value
**Turbidity**	NTU	1.2±0.03
**NPOC**	$mg \cdot L^{-1}$	7.3±0.40
**Conductivity**	$\mu S \cdot cm^{-1}$	69.9±1.9
**Na^{+}**	$mg \cdot L^{-1}$	8.42±0.08
**K^{+}**	$mg \cdot L^{-1}$	1.58±0.01
**Mg^{+}**	$mg \cdot L^{-1}$	0.95±0.01
**Ca^{+}**	$mg \cdot L^{-1}$	2.67±0.03
**Cl^{-}**	$mg \cdot L^{-1}$	7.83±0.13
**SO_4^{2-}**	$mg \cdot L^{-1}$	0.73±0.10

References:

(1) Casado, N.; Hernández, G.; Veloso, A.; Devaraj, S.; Mecerreyes, D.; Armand, M. PEDOT Radical Polymer with Synergetic Redox and Electrical Properties. *ACS Macro Lett.* **2016**, *5* (1), 59–64. https://doi.org/10.1021/acsmacrolett.5b00811.

(2) Schwartz, P. O.; Pejic, M.; Wachtler, M.; Bäuerle, P. Synthesis and Characterization of Electroactive PEDOT-TEMPO Polymers as Potential Cathode Materials in Rechargeable Batteries. *Synth. Met.* **2018**, *243* (June), 51–57. https://doi.org/10.1016/j.synthmet.2018.04.005.

(3) Cao, F.; Wang, L.; Ren, X.; Sun, H. Synthesis of a Perfluorooctanoic Acid Molecularly Imprinted Polymer for the Selective Removal of Perfluorooctanoic Acid in an Aqueous Environment. *J. Appl. Polym. Sci.* **2016**, *133* (15), 1–10. https://doi.org/10.1002/app.43192.

(4) Yu, Q.; Deng, S.; Yu, G. Selective Removal of Perfluorooctane Sulfonate from Aqueous Solution Using Chitosan-Based Molecularly Imprinted Polymer Adsorbents. *Water Res.* **2008**, *42* (12), 3089–3097. https://doi.org/10.1016/j.watres.2008.02.024.

(5) Cao, F.; Wang, L.; Tian, Y.; Wu, F.; Deng, C.; Guo, Q.; Sun, H.; Lu, S. Synthesis and Evaluation of Molecularly Imprinted Polymers with Binary Functional Monomers for the Selective Removal of Perfluorooctanesulfonic Acid and Perfluorooctanoic Acid. *J. Chromatogr. A* **2017**, *1516*, 42–53. https://doi.org/10.1016/j.chroma.2017.08.023.

(6) U.S. EPA. Definition and Procedure for the Determination of the Method Detection Limit—Revision 1.11. *Epa 821-R-16-006* **2016**, No. December, 1–8.

(7) Li, Z.; Chen, T.; Cui, F.; Xie, Y.; Xu, W. Impact of Chitosan and Polyacrylamide on Formation of Carbonaceous and Nitrogenous Disinfection By-Products. *Chemosphere* **2017**, *178*, 26–33. https://doi.org/10.1016/j.chemosphere.2017.02.140.
